# Editorial

**Published:** 1839-06-01

**Authors:** 


### THE MEDICAL EXAMINER.

PHILADELPHIA, JUNE 1, 1839.

In the present number will be found a letter from our Parisian correspondent, Dr. Martins; arrangements were made for regular correspondence with Europe at the beginning of the last winter, but, owing to accidental delays, were not completed until recently. We have the pleasure to announce to our readers that we shall furnish them with a series of communications from Dr. Martins and from Dr. Clymer, one of the editors of the Examiner, who is now on his voyage to Europe.

This correspondence is chiefly, but not exclusively, derived from Paris, which is the great centre of medical instruction. It will be rendered more complete by translations of the most important articles from the French journals, and from the Bulletin of the Royal Academy of Medicine, which contains copious reports of the interesting proceedings of that body. These journals are either received by mail, or at stated periods, soon after their publication.

The English journals contain such copious accounts of the progress of medical science in Great Britain, that we cannot do better than present our readers with a condensed summary of the leading articles, and a reprint of the most interesting lectures contained in them. These reprints are furnished by the Examiner very soon after their publication in Great Britain; in some cases we placed articles in the hands of our readers in less than a month after their publication in Great Britain, in other cases within six weeks.

The great facilities, therefore, for obtaining professional information from Europe, have not rendered us indifferent to the importance of drawing more largely on our stock of domestic material. If at times we have been unable to furnish as large a proportion of original matter as we could have desired, we have never forgotten that one of the great objects of the Examiner is to furnish an easy and rapid means of professional communication amongst the physicians of distant parts of the country, and we would again urge upon our medical brethren the advantages which would result to themselves, as well as to the profession in general, if they would avail themselves more frequently of such facilities. Their concurrence would enable us to publish such a collection of documents, relative to the diseases of this country, as would settle many doubtful questions, and render treatment more easy of application, and more sure in its results.

---

What are the causes of the wide-spread ill-health of the present generation of clergymen? This question seems to be just now exciting attention in New England, where the disease, called *clergymen's sore-throat*, is represented to be distressingly prevalent. Several New England physicians have addressed papers on this subject to the Editor of the Boston Medical and Surgical Journal, and most of them unite in the opinion that the practice of the *ultra-temperance* doctrines, together with increased mental and vocal efforts in the service of the legion of societies which have overspread the country, may be set down as the exciting causes of the clerical sufferings. The clergy of the olden time were strangers to the epidemic of this generation. But their avocations were lighter, their habits of living more generous. Their duties were more strictly parochial,—they were not called upon, night after night, to harangue assemblies of abolition, or colonization, or temperance associations; and they felt no scruples of conscience touching the moderate and sober enjoyment of the good things of this life. The parson Adamses, who indulged in tankards and pipes, had no sore throats.

A writer in the Boston Journal, who signs himself *Senex*, and states that he is himself the son of a clergyman, bears the following testimony on the subject:

"Half a century ago, most of our clergymen laboured some, particularly in the time of getting hay. Nearly all made their gardens and cut their wood; and several of them did considerable towards cultivating their own small farms. Their parochial duties were comparatively light; they seldom preaching more than two sermons a week; and from the composition of these they were frequently relieved, by exchanges about once a month, or oftener during summer. Most of them smoked tobacco, and some of them chewed. I recollect but one old minister who took snuff. They all drank cider daily, and when fatigued, generally took some flip or toddy. Very few of them, if any, however, were in the habit of drinking ardent spirit daily. That refreshing drink, small beer made from malt, was considered nearly as necessary as bread in a family. Thus they frequently led very quiet and regular lives, without being exposed to intense excitement of mind, or over fatigue of body. On the whole, they were probably as healthy, and lived as long, as most other classes of the community. My own father lacked only about two months of being eighty-eight years old, when he died.

Within the last twenty years, it would seem that clergymen, in about equal proportions, in our different denominations, have been breaking down by scores, and more particularly as respects their voice. I have been more familiar with with these cases among the Methodists, Episcopalians, and Congregationalists, though I presume there is a proportional number among the Baptists.

There have been some very striking changes in the condition of most of the clergy in New England, since the commencement of the present century. In the first place, their duties are much increased, public opinion requiring of them, in most places, much greater exertion than their predecessors commonly made. The religious meetings, benevolent associations, societies for promoting various objects, and new organizations of almost every description, are, perhaps, ten times as numerous as they were forty years ago. To say that four times as many sermons and addresses are now delivered before a given population, as was formerly the case, would be an estimate, probably, much below the fact. Besides these additional labours, the habits and manner of living of very many clergymen, are essentially changed. Few now have either leisure or land to enable them to attend to agriculture. Most of them have renounced tobacco. When they are fatigued, the slow process of self-restoration is adopted, instead of the expedient of more suddenly reviving the exhausted powers of the system, by the diffusible stimuli. Alcohol in all its forms is proscribed. Few use wine, and most even dispense with cider. Many families have dropped the use of coffee, some of tea, and a considerable number have materially lessened the quantity of animal food. Malt beer, that ancient, harmless beverage, is scarcely known."

